# Diphtheria

**Published:** 1879-01

**Authors:** 


ELMIRA, N. Y., JANUARY, 1879.

The BISTOURY is published Quarterly, upon the 1st of April, July, October and January, at Fifty Cents a year in advance.

### DIPHTHERIA.

Public attention has been so much engrossed with this alarming disease, and so much has been written and said about it that better not have reached the public eye and ear, while the cause and danger of the disease seems to be so imperfectly understood, by the laity, that we are induced to try our hand at throwing some light upon the subject.

We hear it frequently asserted that diphtheria is a disease of comparatively recent origin, that "we did not hear of it until late years." Not so, however: the disease is as old as any of which we have any authentic history. Even Hippocrates, the first great physician, mentions it, and recommended sulphate of copper and honey as a remedy. The disease has appeared several times in Europe as an epidemic, since 1557 to the present time, therefore, is not confined to our own land, as very many suppose.

The editor of the *Saturday Miscellany* believes, "as yet," that it has not been proven contagious. Therein he is in error, and as the public well know that the gentleman referred to would not publish an opinion of so much importance without having given the subject thought and investigation, we hasten to correct his impression of its non-contagiousness, and to assure him that recent investigations, by competent and undoubted authority, reveal the disease to be highly contagious.

Diphtheria is an infectious disease—one that originates through some subtle poison, called miasm, taken into the system, and which poison can reproduce itself indefinitely. A Disease is *endemic* when always present in the same locality; *epidemic*, when it breaks out occasionally; and *contagious* when it can be communicated from one person to another by direct or indirect contact. Diphtheria has all these attributes.

Dr. Oertel, of Munich, has given much time to a patient and exhaustive investigation of the disease. So thorough have been his experiments and observations that he has given the little, vegetable organisms, called bacteira, names, after having divided them into four genera, and describing their peculiarities. The species, named *micrococcus*, are most abundant in the diphtheritic membrane, and the larger their number, the more severe is the disease. If they are placed upon the eye of any animal, the whitish membrane, characteristic of diphtheria, is immediately produced, spreads rapidly over the mucous surfaces of the body, and kills the animal about the fourth or fifth day.

These micrococci, the germs of which we know by the common name "miasm," seem to abound in low, damp places; in localities where vegetable matter is strewn about upon the surface of the ground, and particularly in impure water. The water in wells becomes contaminated with them or their germs, through neighboring cesspools, or from impure surface water that trickles into all wells, to find favorable conditions for their abundant reproduction upon the mucous membranes of our children when they partake of the fluid. Wells, in a city as densely populated as Elmira, *are unsafe.* They should never be used. The danger is greatly enhanced in this city by reason of our imperfect drainage.

The children residing in houses resting close to the ground, or with damp cellars, or where sewer gas escapes into the dwelling through the water or kitchen sink traps, are most endangered to the bacterian influence. Generally, diphtheria can be traced to one or more of the causes mentioned, impure well water, perhaps, being the most frequent. Climate seems to exert no special influence upon the disease, as it is found alike in cold and hot temperatures, pervading every habitable country of the globe.

The disease manifests itself by two sets of symptoms—*local* and *general.*

The local affection makes its appearance upon the mucous membrane of the tonsils or of the soft palate, in the form of a grayish-white deposit, which spreads rapidly in every direction, until mouth, nostrils, throat and wind-pipe become involved. It is thought that it attacks these parts first, because the poisonous germs naturally come in contact with these surfaces whether they are taken in by drinking or by inhalations of poisoned air.

If the spread of the membrane is not checked, the micrococci find their way into the deeper structures of the body, giving rise to the general disease, resembling somewhat the symptoms that accompany the gravest form of typhoid fever.

But what we desire more particularly to call the attention of our readers to is, its contagiousness, that they may exert the utmost care in keeping themselves and their children away from the dwellings where the disease is known to be.

Oertel distinctly says: "Diphtheria is a *miasmatic contagious disease.* When it once has gained a foothold, it becomes widely diffused by contagion. The fact of its *contagiousness* is established, as well by actual cases which occur as by experiment."

Again he says: "The virulence of the contagion is in proportion to the severity of the case from which it comes; the more it is allowed to collect in the room where the patient lies, that is, the less care is bestowed upon ventilation, removal of the expectoration, and cleanliness in general, the more active does it become."

Another author states that diphtheria was communicated to four persons in a family by a son, just returned from college, with diphtheritic membrane in his throat.

Dr. Wiesbauer, in Munich, lost a child that contracted the disease from playing with a canula that had been employed about a patient sick from diphtheria.

But we need not go so far from home to prove its contagiousness:

A prominent physician of Canton, Pa., recently related to us the origin of an epidemic of diphtheria in his town. Thirteen cases occurred in his own practice last winter, every one of which was traced to a pupil who came into the neighborhood from the Mansfield Normal School, where the disease was raging. These thirteen cases were divided among eight families that were widely separated, but had communication either with the boy, who brought the affection to the town, or with a member of some family who had been affected by him.

The contagion can be carried through the air, or upon the clothing of a visitor to an affected house, when it is capable of being spread over the neighborhood to persons susceptible to it.

All exposed to the contagion, fortunately, do not take it. Young children—over a year old—are *most* susceptible. But no person should be permitted to visit a house where diphtheria is raging, nor attend a funeral where a death has occurred from this disease, simply because *he* is not afraid of contracting it. He *may* escape it himself, while he carries the germs of the fatal malady to his innocent little ones, or to those of his neighbor.

What we most need is more thoughtfulness in such matters. No one can be of any possible *use* at a funeral. If you desire to express your sympathy to the afflicted parents who have lost a child from diphtheria or scarlet fever, do so by letter. But for the sake of your children, for protection of the community, and as an evidence of good sense *do* remain away from the infected premises.

Upon the treatment of diphtheria we have nothing to say. The best course to pursue when a member of your family is attacked, is to send early for your trusted family physician. Then place a card upon your door labeled

DIPHTHERIA, and refuse admission to all idle and thoughtless visitors.

Further: Is not this a proper time to take into consideration the best means of disposing of our dead? Is it not conceivable that the cause of the present epidemic of diphtheria might be traced to Woodlawn—our burial place? The cemetery is located upon a gravelly knoll, from fifteen to twenty-five feet higher than the plane of the city. The natural flow of the water from this mound is toward the river and through the wells of the city. Hundreds of decaying, putrid bodies have been drenched by the unusually abundant rains: the ground has not been frozen during the prevalence of the epidemic, so that the percolation has been favored. Is it not possible that our dead have been poisoning the living?

A wholesome, cleanly remedy exists in cremation. Why any one should object to this process —why the slow rotting of the body in the ground, with nasty, squirming worms wriggling through the stinking mass, should be preferred to the cleanly one of burning the body to dust, in a few minutes, does not clearly appear.

Cremation must recommend itself as a cleanly, wholesome and inexpensive mode of disposing of our dead. It will be opposed by those whose business it is to conduct costly funerals, and by the superstitious members of community, but the day is not far distant, we earnestly hope, when every cemetery in the land will possess a cremation furnace, as one of the means of driving pestilence from our midst.

If it be true—and we regard it as not unlikely —that the wells are poisoned from the source mentioned, is it not a powerful argument in favor of disposing of dead bodies in a more rational manner?

Are we forever to *bury* our dead, simply because that has been the recognized and accepted plan for thousands of years? This method was good enough in the days of its origin, when the land was less densely populated, but now, it must seem to every thoughtful person a constant danger to the living.

